# Twenty-Four week Taichi training improves pulmonary diffusion capacity and glycemic control in patients with Type 2 diabetes mellitus

**DOI:** 10.1371/journal.pone.0299495

**Published:** 2024-04-18

**Authors:** Xiaoli Liu, Huan Zhu, Yong Peng, Yaofeng Liu, Xiangrong Shi

**Affiliations:** 1 Department of Physical Education, Xihua University, Chengdu, Sichuan, China; 2 Department of Physical Education, Hubei Minzu University, Enshi, Hubei, China; 3 University of North Texas Health Science Center, Fort Worth, Texas, United States of America; UNAM Facultad de Estudios Superiores Zaragoza: Universidad Nacional Autonoma de Mexico Facultad de Estudios Superiores Zaragoza, MEXICO

## Abstract

This study evaluated the effect of 24-week Taichi training and Taichi plus resistance band training on pulmonary diffusion capacity and glycemic control in patients with Type 2 diabetes mellitus (T2DM). Forty-eight patients with T2DM were randomly divided into three groups: Group A—Taichi training: practiced Taichi 60 min/day, 6 days/week for 24 weeks; Group B—Taichi plus resistance band training: practiced 60-min Taichi 4 days/week plus 60-min resistance band training 2 days/week for 24 weeks; and Group C–controls: maintaining their daily lifestyles. Stepwise multiple regression analysis was applied to predict diffusion capacity of the lungs for carbon monoxide (DLCO) by fasting blood glucose, insulin, glycosylated hemoglobin (HbA1c), tumour necrosis factor alpha (TNF-α), von Willebrand Factor (vWF), interleukin-6 (IL-6), intercellular adhesion molecule 1 (ICAM-1), endothelial nitric oxide synthase (eNOS), nitric oxide (NO), endothelin-1 (ET-1), vascular endothelial growth factor, and prostaglandin I-2. Taichi with or without resistance band training significantly improved DLCO, increased insulin sensitivity, eNOS and NO, and reduced fasting blood glucose, insulin, HbA1c, TNF-α, vWF, IL-6, ICAM-1, and ET-1. There was no change in any of these variables in the control group. DLCO was significantly predicted (R^2^ = 0.82) by insulin sensitivity (standard-β = 0.415, P<0.001), eNOS (standard-β = 0.128, P = 0.017), TNF-α (standard-β = -0.259, P = 0.001), vWF (standard-β = -0.201, P = 0.007), and IL-6 (standard-β = -0.175, P = 0.032) in patients with T2DM. The impact of insulin sensitivity was the most important predictor for the variation of DLCO based on the multiple regression modeling. This study demonstrates that 24-week Taichi training and Taichi plus resistance band training effectively improve pulmonary diffusion capacity and blood glycemic control in patients with T2DM. Variation of DLCO is explained by improved insulin sensitivity and endothelial function, and reduced inflammatory markers, including TNF-α, vWF, and IL-6.

## Introduction

Diabetes mellitus (DM) is a chronic disease resulting from impaired secretion of insulin and/or impaired response to insulin. Main subtypes of DM include Type I DM (T1DM) which presents in children or adolescents, and Type II DM (T2DM) which is more prevalent in middle-aged or older adults and has a more complex pathological process as compared to T1DM [1, 2]. It has been estimated that over 5.37 million people worldwide had DM in 2021; among them approximately 90% were T2DM [2, 3]. DM is regarded as one of the major threats to public health, which seems to preferentially cause the damage to organs with the dense microvascular circulation. This explains high prevalence of retinopathy [4], nephropathy [5], and pulmonary capillary membrane dysfunction [6] in DM patients. It seems that the lung becomes a primary target organ for DM-impaired pulmonary function [7] as a result of pulmonary fibrotic changes [8] and microvasculature disorders [9–12]. Glycosylation of pulmonary elastin and collagen fibers stiffens the lungs and causes emphysema-like reduction in alveolar surface area and impairment of vascular diffusion and elastic recoil in young patients with DM [13]. Borst et al reported that diabetes, in the absence of overt pulmonary disease, is associated with a modest impaired pulmonary function [7]. Saini et al [14] demonstrated that patients with T2DM had a reduction of almost 10% in peak expiratory flow (PEF) and FEF25–75%, which indicated that there was impairment in the large and small airways. A study by Díez-Manglano et al [3] showed that all of the pulmonary function test results were decreased in the patients with T2DM. Recent study reported that patients with T1DM had a reduced pulmonary diffusion capacity evident by a low blood oxygen level [15]. Since pulmonary diffusion capacity is a vital function of the lung and DM is known as a risk factor that damages the pulmonary gas exchange membrane, we hypothesized that improved glycemic control may help enhance pulmonary diffusion capacity in patients with T2DM.

Taichi is a traditional Chinese meditative movement combining low- to moderate-intensity physical exercise with slow and deep breathing [16]. Practicing Taichi is reportedly to increase vital capacity and maximal oxygen uptake in middle-aged and elderly adults [17, 18] and improve inflammation profiles and pulmonary function in children with mild asthma [19]. Aerobic exercise training, such as Taichi, has been shown to improve arterial compliance and capillary density [20]. However, the effect of Taichi training on blood glycemic control remains to be elusive, despite the view that practicing Taichi may be beneficial for the management of blood levels of glucose and glycosylated hemoglobin (HbA1c) in patients with T2DM [21]. Previously, Zhang and Fu [22] reported that Taichi training 60 min/day, 5 days a week for 14 weeks significantly improved glycemic control in patients with T2DM. Tsang et al [23], however, did not find a significant change in insulin resistance or HbA1c in patients with T2DM following Taichi training 60 min/day, 2 days a week for 16-weeks. Li et al [24] reported that a change in HbA1c after practicing Taichi 60 min/day, 5 days/week for 12 weeks was worse as compared with the no-training control. On the other hand, Chen et al [25] observed a marginal decrease in HbA1c (from 8.9 ± 2.7% to 8.3 ± 2.2%, P = 0.064) with 60 min/day, 3 days/week for 12 weeks. It is likely that the inconsistence of these prior studies may be related to the difference in training frequency or volume, or the adherence to the training regimen [26], or the patients with unstable baseline condition and/or incoherent history duration with T2DM [24]. Thus, study with adequate training volume, well-controlled training regimen and/or more coherent patients with stable T2DM history is needed to provide the conclusive impact of Taichi training on T2DM. Furthermore, currently, there is a lack of studies regarding the effect of Taichi training on pulmonary diffusion capacity in patients with T2DM.

The present study applied Taichi training with 60 min/day, 6 days/week for 24 weeks to investigate the beneficial effect on pulmonary diffusion capacity and blood glycemic control, and moreover, the association of pulmonary diffusion capacity with blood levels of glucose, insulin, HbA1c, inflammatory markers, and endothelium-derived factors in older patients with T2DM. Since resistance band training is reportedly to reduce HbA1c and increase insulin sensitivity [27], and since strength exercise combined with or without aerobic exercise reduce risk for T2DM [28], provide anti-inflammatory effect [29], and exhibited better respiratory function [30], we postulated that Taichi concurrently combined with resistance band training would likely afford additional beneficial effect on lung diffusion function in patients with T2DM. Therefore, another aim of the study was to compare the impact of Taichi training alone vs Taichi plus resistance band training on pulmonary diffusion capacity and blood glycemic control in T2DM.

## Materials and methods

### Study participants

Fifty-eight (29 women) patients with T2DM between 58 and 69 years old voluntarily were enrolled in this study after giving a written, informed consent and passing a physical examination. The study protocol was reviewed and approved by the ethical committee of the Hubei Minzu University (Protocol No. 2021–033). Participants were recruited through the advertisement, recommendation by the friend/family member, and/or referral by the physician. The participants were diagnosed with T2DM for a time period of over 4 to 12 years and took prescribed oral hypoglycemic medications without insulin. They had fasting blood glucose over 7.0 mmol/L (or 126 mg/dL) with HbA1c exceeding 6.5%. The exclusion criteria included (1) glycated hemoglobin (HbA1c) ≥9%, (2) taking insulin, (3) clinical findings of diabetic micro- or macrovascular complications, (4) smoking currently, (5) inability to walk for exercise, (6) being involved in other instructor-led exercise training program, (7) anemia or qualitative hemoglobin abnormality, (8) chronic obstructive pulmonary diseases, (9) medical conditions for which the exercise program might be contraindicated, and (10) pulmonary infection (during the treatment or recovery period).

Taichi training and Taichi with resistance band training interventions lasted approximately 8 months from May through December 2021. During or after the training interventions and data collections, all authors had access to the data with non-identifiable subjects’ information. Table 1 summarizes the basic physical and physiological characteristics of the participants.

**Table 1:** Basic physical and physiological characteristics.

	Group A	Group B	Group C	*P* value
	(n = 16)	(n = 16)	(n = 16)	
Age (years)	60.6 ± 5.7	61.4 ± 3.4	61.3 ± 4.9	0.893
Weight (kg)	69.1 ± 8.4	69.0 ± 9.6	67.9 ± 7.0	0.890
Height (m)	1.62 ± 0.07	1.62 ± 0.06	1.60 ± 0.07	0.835
BMI (kg/m^2^)	26.5 ± 3.5	26.2 ± 3.4	26.3 ± 2.2	0.977
Number with BMI >30	3	3	1	0.512
HR (bpm)	80 ± 11	78 ± 7	80 ± 13	0.792
SBP (mmHg)	123 ± 18	124 ± 12	122 ± 16	0.957
Number with SBP >140	5	3	4	0.582
DBP (mmHg)	86 ± 9	83 ± 10	79 ± 11	0.205
Number with DBP >90	7	6	3	0.715
Years with T2DM	7.5 ± 2.0	7.8 ± 2.0	7.4 ± 2.3	0.868
DLCO (ml/min/mmHg)	6.91 ± 0.63	6.86 ± 0.54	7.05 ± 0.83	0.727

Group A: Taichi training; Group B: Taichi plus resistance band training; Group C: control; BMI: body mass index; HR: heart rate; SBP: systolic blood pressure; DBP: diastolic blood pressure; T2DM: type II diabetes mellitus; DLCO: diffusion capacity of the lungs for carbon monoxide. P values indicate significance level. One-way ANOVA was tested for the difference in continuous variables. Chi square test (X^2^) was applied to examine the difference in the number of subjects whose BMI, SBP and DBP were over 30 kg/m^2^, 140 mmHg and 90 mmHg, respectively. All variables are not significantly different among the three groups. Data are group mean ± standard deviation (SD) of the mean.

### Study design and interventions

The subjects were randomly assigned to three groups through computer-generated random numbers: Group A—Taichi training (n = 20, 10 women), Group B—Taichi plus resistance band training (n = 20, 10 women), and Group C—the controls (n = 18, 9 women). Before the initiation of the training program, participants in Group A and Group B were allowed to be familiarized with the Taichi exercise–Yang’s style 24 movements for up to 2 weeks [31]. The sample size was estimated based on a previous study which seemed adequately powered with a total of 20 subjects for the Taichi training and control groups [22]. The participants in Group A practiced Taichi 60 min per day, 6 days a week for 24 weeks. The participants in Group B practiced Taichi 60 min a day, 4 days a week; in addition, they trained with resistance band 60 min a session or day, 2 days a week for 24 weeks. The resistance band exercises consisted of shoulder flexion, shoulder abduction, triceps extension, biceps curl, squat, forward lunge, hip adduction/abduction, hip extension by using elastic bands (Lining, China). Intensity of the resistance band exercise was 40–50% 1 repetition maximum (1RM), 2 sets of 8–12 repetitions [32]. The training load was increased when the subject was able to do 1–2 repetitions more than prescribed by adjusting band stretch amplitude and grip width. The participants in Group C just received regular treatment for their diabetes mellitus at the clinics and were requested to maintain their lifestyle daily without doing exercise training and to record the activity daily and to submit the recorded logs weekly. All training sessions were led by the experienced instructors and conducted in the designated sites on the campus and in the city park. Participants’ heart rate was monitored by Polar monitor one training session or day per week. The averaged heart rate was between 95 to 105 beats/min during practicing Taichi and between 95 to 115 beats/min during band resistance training. Meanwhile, all participants were requested to maintain their daily routine, medications, and normal dietary habits during the entire study period. Four patients (2 men) in Group A and Group B, respectively, did not completed the training programs. Two participants in Group C (1 man) did not complete the post-intervention assessments. Therefore, 48 subjects, 16 subjects in each group, were included in the data analysis. There was no any adverse or unexpected event occurred in the study, no deviation from the approved study protocol.

### Study outcomes

The primary outcome was pulmonary diffusion capacity assessed by the carbon monoxide diffusion. The secondary outcomes included fasting blood glucose, insulin and HbA1c levels. Inflammatory markers and endothelium-derived factors were considered as the tertiary outcomes.

#### Testing for pulmonary diffusion capacity

The diffusion capacity of the lungs for carbon monoxide (DLCO) was determined using single-breath method as previously described [33] in the respiratory division at the affiliated hospital of the Hubei Minzu University. The subject performed this testing in a seated position and breathed quietly with a nose clip in place (Power Cube Boay System; Ganshorn, Germany). The subject was then asked to give a rapid inhalation up to vital capacity following an exhalation up to residual volume, and to hold the breath for 10 sec. The test gas contained 0.3% carbon monoxide and 0.3% inert gas with 21% oxygen balance nitrogen [33]. Before the testing, all participants were required to be familiarized with the maneuver by practice with a couple of trials without breathing carbon monoxide. All participants’ DLCO tests were conducted before (baseline) and after 24-week interventions by the same physicians who oversaw the respiratory function assessment unit in the division.

#### Testing of blood markers

Fasting venous blood samples (>8 hours from the last meal) were taken by trained medical staff in the Affiliated Hospital of the Hubei Minzu University. Fasting blood glucose (FBG) was tested using hexokinase method (Labospect 008 AS, Hitachi, Japan) with Glucose Assay Kit (No. 20172400968, Life Origin Biotech, Wuhan, China). Insulin was tested using ChemiLuminescence method (Advia Centaur XP Immunoassay System, Siemens, Germany) with Insulin Assay Kit (No. 20152400847, Life Origin Biotech, Wuhan, China). HbA1c was determined using high performance liquid chromatography (Hemoglobin Analysis Instrument I-19, Lifotronic, Shenzhen, China) with HbA1c Assay Kit (No. 20152401314, Life Origin Biotech, Wuhan, China). All these assays were processed single-blinded in the Hubei Minzu University immediately. Insulin sensitivity (Insulin S) was calculated [34] from the equation:

$$Insulin\;S=1/[lg\;FBG\;\left(mmol/l\right)+\text{lg}\;Insulin\;(\mu IU/ml)].$$

Inflammatory markers tumour necrosis factor alpha (TNF-α), interleukin-6 (IL-6), intercellular adhesion molecule 1 (ICAM-1), and von Willebrand factor (vWF) were assessed by enzyme-linked immunosorbent assay (ELISA) (No. H052-1-1, No. H007-1-1, No. H065-1-1 and No. H185-1-1, DG5033A ELISA Instrument, East China Electronics Co., Nanjing, China). Endothelial nitric oxide synthase (eNOS), vascular endothelial growth factor (VEGF), prostaglandin I-2 (PGI-2), and endothelin-1 (ET-1) were determined using ELISA (No. H195-1-1, No. H044-2-1, No. H214-1-1 and No. 0H093-1-1, DG5033A ELISA Instrument, East China Electronics Co., Nanjing, China). Endothelial derived nitric oxide (NO) was tested using nitrate reductase method (No. A12-1-1, ZD-6A Automatic Nitrate Reductase Instrument, China Testing Alliance, Beijing, China). All measurements were conducted single-blinded in the collaborator laboratory at Nanjing Jiancheng Bioengineering Institute (Nanjing, China) using the Assay Kits and protocols provided by the Nanjing Jiancheng Bioengineering Institute. The plasma samples for inflammatory markers and endothelium-derived factors before and after interventions were stored in -80°C freezer before the assays and analyzed at once after de-freezing of the samples.

### Statistical analysis

Normality distribution was verified using the Shapiro-Wilk test. All baseline variables and changes in DLCO passed the normality test. One-way ANOVA was tested for the difference in continuous baseline data and chi-square test was conducted for the difference in categorical baseline data among the three groups. Paired t-test was applied to determine the significance of the changes (i.e., after—before) following 24-week interventions. Two-factor ANOVA was performed to test the significance of the group factor and time factor (before vs after). Bonferroni’s post-hoc analysis was conducted if the main factor showed a significance at *P* < 0.05. A stepwise multiple regression analysis was applied to predict the subjects’ pulmonary perfusion capacity (i.e., DLCO). The predictors in the regression modeling included all the variables of blood glycemic control related indexes including insulin sensitivity, inflammatory markers and endothelium-derived factors. In addition, Pearson correlation coefficients were analyzed to determine the association of DLCO with these variables. Data analysis was carried out using SPSS 25.0 software. Group data for all continuous variables were reported as group mean ± standard deviation (SD) of the mean. A *P* value of 0.05 or less was considered statistically significant.

## Results

### Taichi on physical characteristics

Although baseline physical characteristics were not different among the groups (Table 1), body weight was significantly decreased after 24-week interventions in Group A (-1.28 ± 2.32 kg, P = 0.044) and Group B (-2.31 ± 2.64 kg, P = 0.005). The decreases in weight were not different between the training interventions with Taichi only (Group A) vs Taichi plus resistance band training (Group B). There was no significant change in weight in Group C (0.55 ± 1.50 kg, P = 0.152). Resting arterial pressure and heart rate were not significantly changed in all groups.

### Taichi on pulmonary diffusion capacity

**Fig 1:**
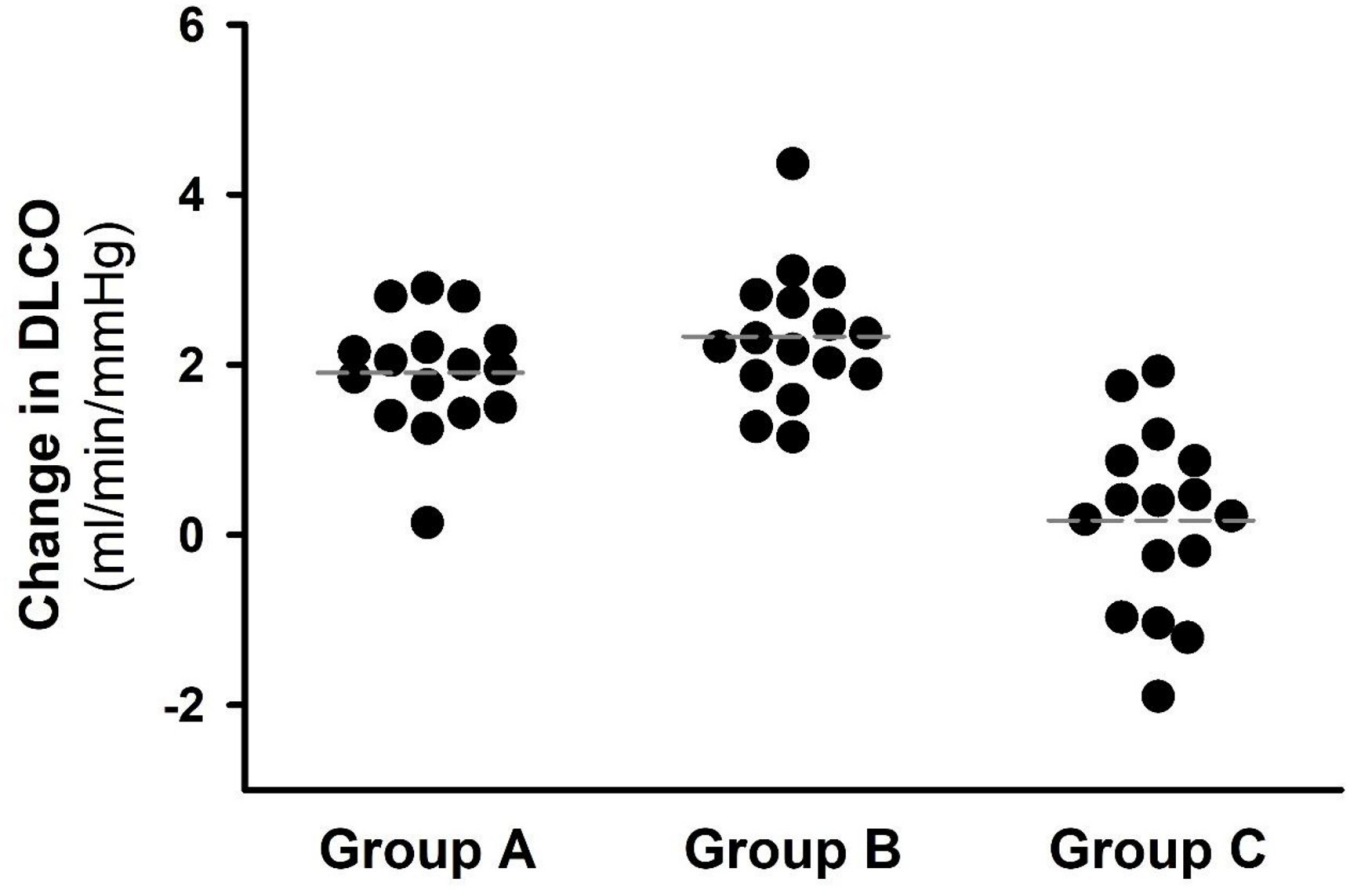
Change in pulmonary diffusion capacity after 24-week interventions. Diffusion capacity of the lungs for carbon monoxide (DLCO) was significantly improved (P < 0.001) in Group A and Group B. There was no DLCO change in Group C. Black dots denote the individual data and the dashed gray lines indicate the group mean.

There was no difference in baseline DLCO among the groups (Table 1). Taichi without (Group A) and with resistance band training (Group B) significantly increased DLCO to 8.81 ± 0.54 and 9.38 ± 0.29 ml/min/mmHg, respectively. There was no change in DLCO in Group C before vs after (7.03 ± 0.79 ml/min/mmHg). Fig 1 illustrates the changes in DLCO after 24-week interventions. The increases in DLCO in both Group A (1.90 ± 0.17 ml/min/mmHg) and Group B (2.33 ± 0.20 ml/min/mmHg) were significant (P < 0.001), which were not different between Group A and Group B. There was no DLCO change in Group C (0.17 ± 0.27, P = 0.534).

### Taichi on glycemic control, inflammatory markers and endothelial-derived factors

Fasting blood glucose was significantly and similarly reduced following 24-week Taichi without (Group A) or with resistance band training (Group B). There was no change in Group C, suggesting that the participants had a stable state with T2DM (Table 2). In parallel with fasting blood glucose, insulin levels were significantly reduced and the estimated insulin sensitivity were significantly enhanced in both Group A and Group B, with no change in Group C. Furthermore, HbA1c were decreased after the 24-week interventions, indicating an improved blood glycemic control associated with Taichi training in Group A and Group B. There was no difference in any of these variables between Group A and Group B (Table 2).

**Table 2:** Glucose metabolic index, inflammatory markers and endothelium-derived factors.

		Time	Group A	Group B	Group C	*P* value	
			(n = 16)	(n = 16)	(n = 16)	Group	Time
**Glucose Metabolic Index**	**Glucose (mmol/L)**	**Before**	8.05±0.52	8.19±0.75	8.12±0.49	0.009	0.002
		**After**	7.40±0.77*	7.27±0.6*^#^	8.21±0.52		
	**Insulin (μIU/ml)**	**Before**	10.15±2.75	9.61±1.59	9.77±2.6	0.065	0.002
		**After**	8.11±1.54*	7.53±1.08*	9.86±2.49		
	**HbA1c (%)**	**Before**	7.46±1.13	7.50±1.06	7.40±0.74	0.099	0.002
		**After**	6.57±0.9*	6.45±0.97*	7.47±0.77		
	**Insulin S (unit)**	**Before**	0.53±0.03	0.53±0.02	0.53±0.03	0.003	0.001
		**After**	0.57±0.03*	0.58±0.02*^#^	0.53±0.03		
**Inflammatory Markers**	**TNF-α (pg/ml)**	**Before**	10.1±1.0	9.6±1.2	9.4±1.0	0.001	0.001
		**After**	7.0±0.6*	6.8±0.5*	9.3±0.8		
	**IL-6 (pg/ml)**	**Before**	5.27±0.72	5.20±0.9	5.40±0.6	0.001	0.001
		**After**	3.38±0.57*	2.80±0.6*^#^	5.10±0.7		
	**ICAM-1 (ng/ml)**	**Before**	359±39	344±39	354±41	0.001	0.001
		**After**	322±38*	286±35*^#^	357±43		
	**vWF (%)**	**Before**	176±13	173±11	180±9	0.001	0.001
		**After**	149±9*	136±10*^#^	177±12		
**Endothelium-derived Factors**	**eNOS (U/ml)**	**Before**	6.74±0.74	7.08±0.61	6.96±0.92	0.013	0.002
		**After**	7.55±0.79	7.93±0.80*^#^	6.85±0.91		
	**NO (μmol/ml)**	**Before**	33.2±4.9	33.4±4.9	32.9±4.7	0.010	0.004
		**After**	37.9±5.1*	38.8±4.7*^#^	31.9±6.2		
	**VEGF (ng/L)**	**Before**	165.7±26.4	171.1±21.1	173.8±27.5	0.625	0.423
		**After**	192.2±23.0	201.4±24.8	169.8±9.2		
	**PGI-2 (ng/L)**	**Before**	99.5±12.3	97.9±10.8	96.3±14.5	0.136	0.052
		**After**	103.5±11.4	97.8±10.3	98.4±14.4		
	**ET-1 (ng/ml)**	**Before**	57.7±7.2	58.0±5.7	58.4±9.2	0.033	0.007
		**After**	51.8±8.3*	50.4±4.0*	59.1±9.0		

Group A: Taichi training; Group B: Taichi plus resistance band training; Group C: control; HbA1c: glycosylated hemoglobin; Insulin S: insulin sensitivity; TNF-α: tumour necrosis factor alpha; IL-6, interleukin-6; ICAM-1: intercellular adhesion molecule 1; vWF: von Willebrand Factor; eNOS: endothelial nitric oxide synthase; NO: nitric oxide; VEGF: vascular endothelial growth factor; PGI-2: prostaglandin I-2; ET-1: endothelin-1. Paired t-test was applied to determine the significance of the changes (after–before) following 24-week interventions. Two-factor ANOVA was performed to test the significance of group factor and time factor (before vs after). Bonferroni’s post-hoc analysis was conducted if the main factor showed a significance, i.e., P < 0.05.

* denotes a significant difference between the baseline and post-intervention within the group and

^#^ indicates a significant difference compared with Group C according to post-hoc analysis. There are no differences in all baseline (Before) variables between the groups. There is no difference in any of these variables between Group A and Group B after 24-week interventions. Data are group mean ± standard deviation (SD) of the mean.

Blood inflammatory markers TNF-α, IL-6, ICAM-1, and vWF were all significantly and similarly decreased after 24-week Taichi training in combination with or without resistance band training; these inflammatory markers were not changed in Group C (Table 2). Furthermore, Taichi interventions with or without resistance band training significantly increased eNOS and NO, and reduced ET-1 level. However, VEGF and PGI-2 were not significantly altered by Taichi interventions with or without resistance band training (Table 2). There was no difference in any of these variables between Group A and Group B following 24-week interventions. There were no changes in these variables occurred in Group C.

### Factors determining pulmonary diffusion capacity

Stepwise multiple regression analysis revealed that 5 predictors or contributors explained 82% of variations of DLCO (Table 3). Among these predictors, insulin sensitivity was the most important factor (standardized-β = 0.415). Insulin sensitivity and eNOS (standardized-β = 0.128, P = 0.017) were positively, whereas inflammatory markers TNF-α (standardized-β = -0.259, P = 0.001), vWF (standardized-β = -0.201, P = 0.007), and IL-6 (standardized-β = -0.175, P = 0.032) were negatively associated with DLCO based on multiple regression modeling. Among the inflammatory markers, TNF-α was more negatively contributed to the impact on DLCO as compared with vWF and IL-6 (Table 3). The order of these 5 predictors was insulin sensitivity, TNF-α, vWF, eNOS, and IL-6 according to the P value and absolute magnitude of standardized regression coefficient (Table 3). Table 4 summarizes the correlations of the changes in DLCO with the changes in various levels of blood glycemic control, inflammatory markers, and endothelium-derived factors. The changes in DLCO were correlated positively with the changes in insulin sensitivity, eNOS and NO, and negatively with the changes in fasting blood glucose, insulin, HbA1c, TNF-α, vWF, IL-6, ICAM-1, and ET-1.

**Table 3:** Predictors for pulmonary diffusion capacity for carbon monoxide.

Predictors	beta	Std beta	t	P	R^2^	Adj R^2^
**Insulin S**	13.767	0.415	7.529	<0.001	0.912	0.820
**eNOS**	0.178	0.128	2.450	0.017		
**IL-6**	-0.165	-0.175	-2.188	0.032		
**vWF**	-0.012	-0.201	-2.784	0.007		
**TNF-α**	-0.195	-0.259	-3.509	0.001		

Predictors that contributed significantly to the subjects’ diffusion capacity of the lungs for carbon monoxide were determined by a stepwise multiple regression analysis. Std beta: standardized regression coefficient; adj R^2^: adjusted determination coefficient based on the multiple linear regression model. Insulin S: insulin sensitivity; eNOS: endothelial nitric oxide synthase; IL-6: interleukin-6; vWF: von Willebrand Factor; TNF-α: tumor necrosis factor alpha.

**Table 4:** Pearson correlation coefficients and *P* values among the changes in variables (n = 48).

	**Insulin S**	**Glucose**	**Insulin**	**HbA1c**	**TNF-α**	**vWF**	**IL-6**	**ICAM-1**	**eNOS**	**NO**	**ET-1**	**VEGF**
**DLCO**	0.680	-0.479	-0.482	-0.575	-0.577	-0.609	-0.614	-0.572	0.667	0.611	-0.500	0.416
	< .0001	0.0006	0.0005	< .0001	< .0001	< .0001	< .0001	< .0001	< .0001	< .0001	0.0003	0.0033
**Insulin S**		-0.821	-0.776	-0.789	-0.660	-0.669	-0.752	-0.706	0.672	0.644	-0.636	0.506
		< .0001	< .0001	< .0001	< .0001	< .0001	< .0001	< .0001	< .0001	< .0001	< .0001	0.0002
**Glucose**			0.489	0.696	0.466	0.557	0.503	0.604	-0.437	-0.401	0.388	-0.315
			0.0004	< .0001	0.0009	< .0001	0.0003	< .0001	0.0019	0.0048	0.0064	0.0293
**Insulin**				0.649	0.604	0.519	0.585	0.580	-0.664	-0.504	0.497	-0.427
				< .0001	< .0001	0.0002	< .0001	< .0001	< .0001	0.0003	0.0003	0.0025
**HbA1c**					0.645	0.637	0.635	0.714	-0.657	-0.502	0.523	-0.393
					< .0001	< .0001	< .0001	< .0001	< .0001	0.0003	0.0001	0.0058
**TNF-α**						0.573	0.619	0.543	-0.63	-0.581	0.522	-0.442
						< .0001	< .0001	< .0001	< .0001	< .0001	0.0001	0.0017
**vWF**							0.654	0.549	-0.529	-0.559	0.491	-0.479
							< .0001	< .0001	0.0001	< .0001	0.0004	0.0006
**IL-6**								0.576	-0.693	-0.603	0.555	-0.493
								< .0001	< .0001	< .0001	< .0001	0.0004
**ICAM-1**									-0.583	-0.457	0.473	-0.392
									< .0001	0.0011	0.0007	0.0058
**eNOs**										0.480	-0.521	0.504
										0.0006	0.0001	0.0003
**NO**											-0.655	0.599
											< .0001	< .0001
**ET-1**												-0.480
												0.0006

DLCO: diffusion capacity of the lungs for carbon monoxide; Insulin S: insulin sensitivity; HbA1c: glycosylated hemoglobin; TNF-α: tumor necrosis factor alpha; vWF: von Willebrand Factor; IL-6, interleukin-6; ICAM-1: intercellular adhesion molecule 1; eNOS: endothelial nitric oxide synthase; NO: nitric oxide; ET-1: Endothelin-1; VEGF: vascular endothelial growth factor. Change is the difference between after and before. Change in prostaglandin I-2 data is not included because of its insignificant correlation with other variables.

## Discussion

This study was among the first to demonstrate that 24-week Taichi training alone or Taichi plus resistance band training significantly improved pulmonary diffusion capacity in patients with T2DM. In addition, our data confirmed that 24-week Taichi training significantly improved blood glycemic control by increasing insulin sensitivity and decreasing fasting blood glucose, HbA1c and insulin in T2DM. The improved pulmonary diffusion capacity in T2DM was significantly explained by enhanced insulin sensitivity, increased eNOS, and reduced inflammatory markers TNF-α, vWF, and IL-6 following 24-week Taichi training. Among these factors, insulin sensitivity was the most important predictor for the improved pulmonary diffusion capacity in patients with T2DM.

### Taichi improved pulmonary diffusion capacity in T2DM

Diffusion capacity of the lungs for carbon monoxide is a sensitive measure of pulmonary diffusion capacity and commonly applied to assess the function of the transfer of gas across the respiratory membrane between the alveoli and capillaries of the lungs [35, 36]. It is reported that DLCO in patient with T2DM is significantly impaired, which is likely associated with the thickening of the gas exchange membrane of the lungs [6], resulting from chronic hyperglycemia [37, 38], dyslipidemia and insulin resistance [39]. Possible pathological processes associated with impaired pulmonary function in T2DM may include non-enzymatic glycosylation of lung elastin structures, which could stiff the lung [40] and cause a restrictive impact on ventilation; and diabetes-induced microvascular complications, which could impair the diffusion [37, 41] and diminish gas exchange. It is reported that lung function assessed by forced vital capacity and forced expiratory volume in 1 sec (FEV-1) are inversely correlated with HbA1c [42–44] and DLCO is significantly impaired in patients with T2DM [44]. Furthermore, patients who have poor glycemic control with HbA1c >7% show lower DLCO or the ratio of DLCO to alveolar ventilation compared to the counterparts with HbA1c <7% [44].

Our data suggested that DLCO could be significantly and similarly improved following 24-week Taichi training or Taichi combined with resistance band training in patients with T2DM (Fig 1). There was no change in DLCO in the patients of Group C. It is known that pulmonary functional capacity and microvascular reserve, reflecting lung volume, alveolar perfusion, and capillary recruitment, are diminished with impaired glycemic control [36]. Slow and deep breathing during practicing Taichi may increase alveolar ventilation and help recruitment more air space. In addition, deep breathing may decrease the thoracic pressure and help facilitate venous return and thus, cardiac output and pulmonary perfusion. These functional responses to practicing Taichi likely help increase the diffusion surface area of the gas exchange membrane and enhance the coupling of ventilation-perfusion of the lung. Long-term practicing Taichi is reportedly to increase vital capacity, cardiac index, and maximal oxygen uptake in older adults [18]. Furthermore, muscle movements during practicing Taichi likely increase insulin sensitivity which may help facilitate pulmonary perfusion function. It has been reported that acute insulin infusion in patients with T2DM improves alveolar-capillary membrane gas exchange and DLCO, independent of the change in vital capacity, FEV-1 or cardiac output [45].

### Taichi improved blood glycemic control in T2DM

Previously, Tsang et al [23] reported no significant improvement in insulin resistance or HbA1c in patients with T2DM following a training program of practicing Taichi 60 min/day, 2 days/week for 16 weeks. Since the patients only trained twice a week, the training volume could be not sufficient to impact blood glycemic control. Furthermore, most of those T2DM patients concurrently had osteoarthritis (84%) and cardiovascular disease (76%), which might diminish the impact of practicing Taichi [23]. Recently, Li et al [24] reported that HbA1c was increased the patients following practicing Taichi 60 min/day, 5 days/week for 12 weeks. Because their patients in the study had the duration of T2DM history from 3 months to 26 years, some of them remained in the unstable state of blood glycemic control and tried different medications including insulin during the intervention, which might cause the unpredictable outcome [24]. Practicing Taichi 60 min/day, 3 days/week for 12 weeks, Chen et al [25] reported a marginal decrease in HbA1c; however, the training regimen significantly improved lipid profiles, and decreased lipid peroxidation marker and C-reactive protein. Our data confirmed the report that Taichi training could significantly improve blood glycemic control [22]. The present study suggested that training volume with practicing Taichi 60 min/day, 6 days/week for 24 weeks in Group A, or 4 days/week plus 2 days with 60 min/day band resistance training for 24 weeks in Group B, was clinically adequate and effective. Both of these regimens could similarly decrease the levels of fasting blood glucose, insulin, and HbA1c, and increased insulin sensitivity for the patients who had stabilized chronic conditions without taking prescribed insulin. There was no significant change in the blood glycemic control in the patients of Group C before vs after.

Our data suggested that improved blood glycemic control, such as decreased levels of fasting blood glucose, insulin and HbA1c, and increased glucose sensitivity following 24-week interventions, was significantly associated with improved DLCO in the patients with T2DM (Table 4). Among these factors, insulin sensitivity was the most important contributor that positively predicted DLCO (Table 3). However, neither HbA1c nor glucose level was included in modeling as a significant contributor. It is likely enhanced insulin sensitivity associated with muscle movements during practicing Taichi could be the driving force to elicit the improved blood glycemic control in the patients with T2DM following 24-week Taichi interventions with or without combination of resistance band training.

### Taichi reduced inflammatory markers in T2DM

Subclinical chronic inflammation is a common feature in the natural course of diabetes. However, practicing Taichi seems to provide anti-inflammation effect. Training programs with Taichi 120 min a week for 3 and 4 months, respectively, are reportedly to show the trend to decrease TNF-α and IL-6 in breast cancer survivors with insomnia [46] and in older adults with insomnia [47]. The present study confirmed that 24-week Taichi training without or with the combination of resistance band training similarly and significantly reduced TNF-α and IL-6 in the patients with T2DM, which were not changed in the control group (Table 2). It is likely that practicing Taichi enhances vagal modulation and calms sympathetic activity [48] which provide inhibitory effects on inflammation [49, 50]. Furthermore, our data suggested that ICAM-1 and vWF were significantly decreased in both Group A and Group B, without change in Group C (Table 2). ICAM-1 is a glycoprotein expressed at a low basal level in immune, endothelial and epithelial cells, but is upregulated in response to inflammatory stimulation [51, 52]. ICAM-1 activates leukocyte rolling and adhesive interaction with the vessel wall and guides leukocyte crossing of the endothelial layer, and predicts development of macrovascular disease in DM [53]. As a plasma glycoprotein, vWF plays an important role in blood clot. However, elevated vWF is considered a pre-inflammatory marker [54] associated with endothelial damage [55]. It is likely that Taichi-induced inhibitory influence on inflammatory markers may contribute to the decreases in ICAM-1 and vWF following the interventions, which could be reinforced by Taichi-improved blood glycemic control and decreased oxidative stress [56].

Furthermore, the present study suggested that pulmonary diffusion capacity was negatively and significantly explained by the inflammatory markers (Table 4). Given all the factors in predicting DCLO, TNF-α was a greater negative predictor, followed by vWF and IL-6, based on multiple regression modeling (Table 3).

### Taichi improved endothelial function in T2DM

Previously, Wang et al [57] observed a significant greater endothelium-mediated cutaneous vasodilation of the leg in Taichi trained (over ≥3 years) elderly men than their age-matched sedentary counterparts, which was similar to the response in a group of younger sedentary men. Shin et al [58] reported that Taichi training 60 min a day for 3 months in the hospital gymnasium significantly enhanced flow-mediated dilation of the brachial artery and reduced arterial stiffness in elderly women with rheumatoid arthritis. Furthermore, a significant increase in plasma NO, and decreases in arterial pressure and low-density lipoprotein cholesterol were observed in patients with essential hypertension following practicing Taichi 60 min a day, 6 days a week for 12 weeks [59]. These prior studies suggested that practicing Taichi could effectively improve endothelial function in elderly adults. Our data confirmed that 24-week Taichi training without or with the combination of resistance band training significantly increased eNOS and NO, and decreased ET-1, although there were no significant changes in VEGF and PGI-2 following the interventions (Table 2). Taichi improved endothelial function is likely mediated by its beneficial impact on blood glycemic control and insulin sensitivity, which may reverse insulin resistance augmented oxidative stress and the damaging effect on endothelial cells. This notion seems to be supported by the popular application of metformin, a non-insulin anti-diabetic medicine, which effectively regulates blood glucose level [60] and meanwhile prevents vascular endothelial dysfunction [61, 62].

The present study also suggested that pulmonary diffusion capacity was positively explained by eNOS, NO, and VEGF, and negatively associated with ET-1 (Table 4). Among all these endothelium-derived factors, however, eNOS was the only significant positive predictor for the variation of DLCO based on multiple regression modeling (Table 3).

### Study limitations and perspectives

There are some study limitations required consideration. First, the participants are older adults, and thus findings may not generalize to younger populations who have better pulmonary diffusion capacity, lower inflammation marker and healthier endothelial function. Second, there is no follow-up assessment in the study and it is unknown how long the Taichi-induced beneficial effects remain in the patients with T2DM. Based on a previous study that the effect of practicing Taichi 120 min weekly for 4 months on reducing inflammatory markers remains 12 months after the end of Taichi training [47], we postulate that the beneficial impacts may last for approximately 1 year. Another study limitation is related to small sample size which is unable to distinguish the difference in Group A vs Group B. Nonetheless, both the primary and secondary outcomes significantly improved after 24-week interventions with Taichi training or Taichi in combination with resistance band training in the present study.

Practicing Taichi provides a nonpharmacological but effective approach for regulating hyperglycemia, reducing inflammatory markers, and improving endothelial function in patients with T2DM. Comparing fasting blood glucose, insulin sensitivity, IL-6, ICAM-1, vWF, eNOS or NO after interventions (Table 2), there is a significant difference in these variables between Group C and Group B; but no any difference observed between Group C and Group A according to *post-hoc* analysis. On the other hand, the efficacy of Taichi training alone vs Taichi plus band resistance training is statistically similar because all factors are not significantly different between Group A and Group B following 24-week interventions. The regimen of practicing Taichi 4 days a week plus resistance band training 2 days a week for 24 weeks does not provide an additional benefit compared to practicing Taichi alone 6 days a week for 24 weeks. Future study is needed to determine the optimal dose of practicing Taichi without including resistance band training, or to focus on the comparison between Taichi training vs resistance band training. Since Taichi training is proved to be safe and effective to improve pulmonary diffusion capacity in older adults with T2DM, we believe that practicing Taichi may be applied as a preventive and therapeutic intervention to combat the COVID-19 pandemic or as a rehabilitation measure for patients recovering from the COVID-19 disease.

## Conclusions

Taichi training or Taichi plus resistance band training can effectively improve pulmonary diffusion capacity and blood glycemic control in patients with T2DM. Variation of DLCO is predicted (adjusted R^2^ = 0.82) by insulin sensitivity, TNF-α, vWF, eNOS, and IL-6. Insulin sensitivity is far more important compared to any other factors, which is likely a driving force to benefit blood glycemic control, anti-inflammation, and endothelial function. Reciprocally, Taichi training reduced inflammatory markers and enhanced eNOS, which reinforce with improved glycemic control, contribute to improved pulmonary diffusion capacity in patients with T2DM.

## Supporting information

S1 Checklist PLOS ONE clinical studies checklist. (DOCX)

S1 File Inclusivity in global research. (PDF)

## References

[1] Daneshgari F, Liu G, Hanna-Mitchell AT. Path of translational discovery of urological complications of obesity and diabetes. American journal of physiology Renal physiology. 2017;312(5):F887–f896. doi: 10.1152/ajprenal.00489.2016 28052873 PMC6109798

[2] Sun H, Saeedi P, Karuranga S, et al. IDF Diabetes Atlas: Global, regional and country-level diabetes prevalence estimates for 2021 and projections for 2045. Diabetes Res Clin Pract. 2022;183:109119. doi: 10.1016/j.diabres.2021.109119 34879977 PMC11057359

[3] Díez-Manglano J, Asìn Samper U. Pulmonary function tests in type 2 diabetes: a meta-analysis. ERJ open research. 2021;7(1). doi: 10.1183/23120541.00371-2020 33569495 PMC7861023

[4] Nathan DM, Genuth S, Lachin J, et al. The effect of intensive treatment of diabetes on the development and progression of long-term complications in insulin-dependent diabetes mellitus. N Engl J Med. 1993;329(14):977–986. doi: 10.1056/NEJM199309303291401 8366922

[5] Effect of intensive therapy on the development and progression of diabetic nephropathy in the Diabetes Control and Complications Trial. The Diabetes Control and Complications (DCCT) Research Group. Kidney Int. 1995;47(6):1703–1720. doi: 10.1038/ki.1995.236 7643540

[6] Wheatley CM, Baldi JC, Cassuto NA, et al. Glycemic control influences lung membrane diffusion and oxygen saturation in exercise-trained subjects with type 1 diabetes: alveolar-capillary membrane conductance in type 1 diabetes. European journal of applied physiology. 2011;111(3):567–578. doi: 10.1007/s00421-010-1663-8 20936482

[7] van den Borst B, Gosker HR, Zeegers MP, et al. Pulmonary function in diabetes: a metaanalysis. Chest. 2010;138(2):393–406. doi: 10.1378/chest.09-2622 20348195

[8] Ban CR, Twigg SM. Fibrosis in diabetes complications: pathogenic mechanisms and circulating and urinary markers. Vascular health and risk management. 2008;4(3):575–596. doi: 10.2147/vhrm.s1991 18827908 PMC2515418

[9] Bai L, Zhang L, Pan T, et al. Idiopathic pulmonary fibrosis and diabetes mellitus: a meta-analysis and systematic review. Respiratory research. 2021;22(1):175. doi: 10.1186/s12931-021-01760-6 34103046 PMC8188656

[10] Navaratnam V, Davis TME, Hubbard R, et al. Incidence and predictors of idiopathic pulmonary fibrosis complicating Type 2 diabetes: the Fremantle Diabetes Study Phase I. Internal medicine journal. 2021;51(2):276–279. doi: 10.1111/imj.15191 33631852

[11] Wang D, Ma Y, Tong X, et al. Diabetes Mellitus Contributes to Idiopathic Pulmonary Fibrosis: A Review From Clinical Appearance to Possible Pathogenesis. Frontiers in public health. 2020;8:196. doi: 10.3389/fpubh.2020.00196 32582606 PMC7285959

[12] Davis WA, Knuiman M, Kendall P, et al. Glycemic exposure is associated with reduced pulmonary function in type 2 diabetes: the Fremantle Diabetes Study. Diabetes care. 2004;27(3):752–757. doi: 10.2337/diacare.27.3.752 14988297

[13] Sandler M, Bunn AE, Stewart RI. Pulmonary function in young insulin-dependent diabetic subjects. Chest. 1986;90(5):670–675. doi: 10.1378/chest.90.5.670 3769567

[14] Saini M, Kulandaivelan S, Bansal VK, et al. Pulmonary Pathology Among Patients with Type 2 Diabetes Mellitus: An Updated Systematic Review and Meta-analysis. Current diabetes reviews. 2020;16(7):759–769. doi: 10.2174/1573399815666190716130324 31333139

[15] Laursen JC, Clemmensen KKB, Hansen CS, et al. Persons with type 1 diabetes have low blood oxygen levels in the supine and standing body positions. BMJ open diabetes research & care. 2021;9(1). doi: 10.1136/bmjdrc-2020-001944 34059524 PMC8169468

[16] Guo Y, Qiu P, Liu T. Tai Ji Quan: An overview of its history, health benefits, and cultural value. Journal of Sport and Health Science. 2014;3(1):3–8.

[17] Tan T, Meng Y, Lyu JL, et al. A Systematic Review and Meta-Analysis of Tai Chi Training in Cardiorespiratory Fitness of Elderly People. Evid Based Complement Alternat Med. 2022;2022:4041612. doi: 10.1155/2022/4041612 35341143 PMC8942636

[18] Sun L, Zhuang LP, Li XZ, et al. Tai Chi can prevent cardiovascular disease and improve cardiopulmonary function of adults with obesity aged 50 years and older: A long-term follow-up study. Medicine (Baltimore). 2019;98(42):e17509. doi: 10.1097/MD.0000000000017509 31626108 PMC6824704

[19] Lin HH, Hung YP, Weng SH, et al. Effects of parent-based social media and moderate exercise on the adherence and pulmonary functions among asthmatic children. The Kaohsiung journal of medical sciences. 2020;36(1):62–70. doi: 10.1002/kjm2.12126 31512391 PMC11896268

[20] Prior SJ, Goldberg AP, Ortmeyer HK, et al. Increased Skeletal Muscle Capillarization Independently Enhances Insulin Sensitivity in Older Adults After Exercise Training and Detraining. Diabetes. 2015;64(10):3386–3395. doi: 10.2337/db14-1771 26068543 PMC4587640

[21] Chao M, Wang C, Dong X, et al. The Effects of Tai Chi on Type 2 Diabetes Mellitus: A Meta-Analysis. Journal of diabetes research. 2018;2018:7350567. doi: 10.1155/2018/7350567 30116744 PMC6079589

[22] Zhang Y, Fu FH. Effects of 14-week Tai Ji Quan exercise on metabolic control in women with type 2 diabetes. Am J Chin Med. 2008;36(4):647–654. doi: 10.1142/S0192415X08006119 18711762

[23] Tsang T, Orr R, Lam P, et al. Effects of Tai Chi on glucose homeostasis and insulin sensitivity in older adults with type 2 diabetes: a randomised double-blind sham-exercise-controlled trial. Age Ageing. 2008;37(1):64–71. doi: 10.1093/ageing/afm127 17965035

[24] Li X, Si H, Chen Y, et al. Effects of fitness qigong and tai chi on middle-aged and elderly patients with type 2 diabetes mellitus. PloS one. 2020;15(12):e0243989. doi: 10.1371/journal.pone.0243989 33332396 PMC7746158

[25] Chen SC, Ueng KC, Lee SH, et al. Effect of t’ai chi exercise on biochemical profiles and oxidative stress indicators in obese patients with type 2 diabetes. J Altern Complement Med. 2010;16(11):1153–1159. doi: 10.1089/acm.2009.0560 20973735

[26] Song R, Ahn S, Roberts BL, et al. Adhering to a t’ai chi program to improve glucose control and quality of life for individuals with type 2 diabetes. Journal of alternative and complementary medicine (New York, NY). 2009;15(6):627–632. doi: 10.1089/acm.2008.0330 19500007

[27] McGinley SK, Armstrong MJ, Boulé NG, et al. Effects of exercise training using resistance bands on glycaemic control and strength in type 2 diabetes mellitus: a meta-analysis of randomised controlled trials. Acta diabetologica. 2015;52(2):221–230. doi: 10.1007/s00592-014-0594-y 24845604

[28] Shiroma EJ, Cook NR, Manson JE, et al. Strength Training and the Risk of Type 2 Diabetes and Cardiovascular Disease. Med Sci Sports Exerc. 2017;49(1):40–46. doi: 10.1249/MSS.0000000000001063 27580152 PMC5161704

[29] Annibalini G, Lucertini F, Agostini D, et al. Concurrent Aerobic and Resistance Training Has Anti-Inflammatory Effects and Increases Both Plasma and Leukocyte Levels of IGF-1 in Late Middle-Aged Type 2 Diabetic Patients. Oxidative medicine and cellular longevity. 2017;2017:3937842. doi: 10.1155/2017/3937842 28713486 PMC5497609

[30] Kalron A, Mahameed I, Weiss I, et al. Effects of a 12-week combined aerobic and strength training program in ambulatory patients with amyotrophic lateral sclerosis: a randomized controlled trial. Journal of neurology. 2021;268(5):1857–1866. doi: 10.1007/s00415-020-10354-z 33388929

[31] Yang SY, Lee HC, Huang CM, et al. Efficacy of Tai Chi-Style Multi-Component Exercise on Frontal-Related Cognition and Physical Health in Elderly With Amnestic Mild Cognitive Impairment. Frontiers in aging. 2021;2:636390. doi: 10.3389/fragi.2021.636390 35822039 PMC9261301

[32] Son WM, Park JJ. Resistance Band Exercise Training Prevents the Progression of Metabolic Syndrome in Obese Postmenopausal Women. Journal of sports science & medicine. 2021;20(2):291–299. doi: 10.52082/jssm.2021.291 34211322 PMC8219266

[33] Graham BL, Brusasco V, Burgos F, et al. 2017 ERS/ATS standards for single-breath carbon monoxide uptake in the lung. The European respiratory journal. 2017;49(1). 10.1183/13993003.00016-2016 28049168

[34] Hao Q, Zheng A, Zhang H, et al. Down-regulation of betatrophin enhances insulin sensitivity in type 2 diabetes mellitus through activation of the GSK-3β/PGC-1α signaling pathway. Journal of endocrinological investigation. 2021;44(9):1857–1868. 33464548 10.1007/s40618-020-01493-1

[35] Fell AK, Notø H, Skogstad M, et al. A cross-shift study of lung function, exhaled nitric oxide and inflammatory markers in blood in Norwegian cement production workers. Occupational and environmental medicine. 2011;68(11):799–805. doi: 10.1136/oem.2010.057729 21297153 PMC3191466

[36] Chance WW, Rhee C, Yilmaz C, et al. Diminished alveolar microvascular reserves in type 2 diabetes reflect systemic microangiopathy. Diabetes Care. 2008;31(8):1596–1601. doi: 10.2337/dc07-2323 18492945 PMC2494655

[37] Kumar A, Bade G, Trivedi A, et al. Postural variation of pulmonary diffusing capacity as a marker of lung microangiopathy in Indian patients with type 2 diabetes mellitus. Indian journal of endocrinology and metabolism. 2016;20(2):238–244. doi: 10.4103/2230-8210.176343 27042422 PMC4792027

[38] Tai H, Wang M-y, Zhao Y-p, et al. Pulmonary Function and Retrobulbar Hemodynamics in Subjects With Type 2 Diabetes Mellitus. Respiratory Care. 2017;62(5):602–614. 28246307 10.4187/respcare.05129

[39] Sinha S, Guleria R, Misra A, et al. Pulmonary functions in patients with type 2 diabetes mellitus & correlation with anthropometry & microvascular complications. The Indian journal of medical research. 2004;119(2):66–71. 15055485

[40] Sandler M. Is the Lung a ’Target Organ’ in Diabetes Mellitus? Archives of Internal Medicine. 1990;150(7):1385–1388. 2196023

[41] Vracko R, Thorning D, Huang TW. Basal lamina of alveolar epithelium and capillaries: quantitative changes with aging and in diabetes mellitus. Am Rev Respir Dis. 1979;120(5):973–983. doi: 10.1164/arrd.1979.120.5.973 507532

[42] Kabeya Y, Kato K, Tomita M, et al. Association of glycemic status with impaired lung function among recipients of a health screening program: a cross-sectional study in Japanese adults. J Epidemiol. 2014;24(5):410–416. doi: 10.2188/jea.je20140016 24998953 PMC4150013

[43] Lee WH, Wu DW, Chen YC, et al. Association of Pulmonary Function Decline over Time with Longitudinal Change of Glycated Hemoglobin in Participants without Diabetes Mellitus. J Pers Med. 2021;11(10). doi: 10.3390/jpm11100994 34683134 PMC8537814

[44] A S, M S, G P, et al. Alveolar Gas Exchange and Pulmonary Functions in Patients with Type II Diabetes Mellitus. J Clin Diagn Res. 2013;7(9):1874–1877. doi: 10.7860/JCDR/2013/6550.3339 24179886 PMC3809625

[45] Guazzi M, Oreglia I, Guazzi MD. Insulin Improves Alveolar-Capillary Membrane Gas Conductance in Type 2 Diabetes. Diabetes Care. 2002;25(10):1802–1806. 12351481 10.2337/diacare.25.10.1802

[46] Irwin MR, Olmstead R, Breen EC, et al. Tai chi, cellular inflammation, and transcriptome dynamics in breast cancer survivors with insomnia: a randomized controlled trial. Journal of the National Cancer Institute Monographs. 2014;2014(50):295–301. doi: 10.1093/jncimonographs/lgu028 25749595 PMC4411534

[47] Irwin MR, Olmstead R, Breen EC, et al. Cognitive behavioral therapy and tai chi reverse cellular and genomic markers of inflammation in late-life insomnia: a randomized controlled trial. Biol Psychiatry. 2015;78(10):721–729. doi: 10.1016/j.biopsych.2015.01.010 25748580 PMC4524803

[48] Motivala SJ, Sollers J, Thayer J, et al. Tai Chi Chih acutely decreases sympathetic nervous system activity in older adults. J Gerontol A Biol Sci Med Sci. 2006;61(11):1177–1180. doi: 10.1093/gerona/61.11.1177 17167159

[49] Hoover DB. Cholinergic modulation of the immune system presents new approaches for treating inflammation. Pharmacol Ther. 2017;179:1–16. doi: 10.1016/j.pharmthera.2017.05.002 28529069 PMC5651192

[50] Irwin MR, Cole SW. Reciprocal regulation of the neural and innate immune systems. Nat Rev Immunol. 2011;11(9):625–632. doi: 10.1038/nri3042 21818124 PMC3597082

[51] Harjunpää H, Llort Asens M, Guenther C, et al. Cell Adhesion Molecules and Their Roles and Regulation in the Immune and Tumor Microenvironment. Front Immunol. 2019;10:1078. doi: 10.3389/fimmu.2019.01078 31231358 PMC6558418

[52] Kong DH, Kim YK, Kim MR, et al. Emerging Roles of Vascular Cell Adhesion Molecule-1 (VCAM-1) in Immunological Disorders and Cancer. Int J Mol Sci. 2018;19(4). doi: 10.3390/ijms19041057 29614819 PMC5979609

[53] Jude EB, Douglas JT, Anderson SG, et al. Circulating cellular adhesion molecules ICAM-1, VCAM-1, P- and E-selectin in the prediction of cardiovascular disease in diabetes mellitus. European Journal of Internal Medicine. 2002;13(3):185–189. doi: 10.1016/s0953-6205(02)00014-6 12020626

[54] Kruger A, Vlok M, Turner S, et al. Proteomics of fibrin amyloid microclots in long COVID/post-acute sequelae of COVID-19 (PASC) shows many entrapped pro-inflammatory molecules that may also contribute to a failed fibrinolytic system. Cardiovasc Diabetol. 2022;21(1):190. doi: 10.1186/s12933-022-01623-4 36131342 PMC9491257

[55] Ladikou EE, Sivaloganathan H, Milne KM, et al. Von Willebrand factor (vWF): marker of endothelial damage and thrombotic risk in COVID-19? Clin Med (Lond). 2020;20(5):e178–e182. doi: 10.7861/clinmed.2020-0346 32694169 PMC7539718

[56] Mendoza-Núñez VM, Arista-Ugalde TL, Rosado-Pérez J, et al. Hypoglycemic and antioxidant effect of Tai chi exercise training in older adults with metabolic syndrome. Clinical interventions in aging. 2018;13:523–531. doi: 10.2147/CIA.S157584 29662308 PMC5892965

[57] Wang J-S, Lan C, Chen S-Y, et al. Tai Chi Chuan Training is Associated with Enhanced Endothelium-Dependent Dilation in Skin Vasculature of Healthy Older Men. Journal of the American Geriatrics Society. 2002;50(6):1024–1030. doi: 10.1046/j.1532-5415.2002.50256.x 12110061

[58] Shin J-H, Lee Y, Kim SG, et al. The beneficial effects of Tai Chi exercise on endothelial function and arterial stiffness in elderly women with rheumatoid arthritis. Arthritis Research & Therapy. 2015;17(1):380. doi: 10.1186/s13075-015-0893-x 26702640 PMC4718020

[59] Pan X, Zhang Y, Tao S. Effects of Tai Chi exercise on blood pressure and plasma levels of nitric oxide, carbon monoxide and hydrogen sulfide in real-world patients with essential hypertension. Clinical and Experimental Hypertension. 2015;37(1):8–14. doi: 10.3109/10641963.2014.881838 24490621

[60] Hostalek U, Gwilt M, Hildemann S. Therapeutic Use of Metformin in Prediabetes and Diabetes Prevention. Drugs. 2015;75(10):1071–1094. doi: 10.1007/s40265-015-0416-8 26059289 PMC4498279

[61] Venu VKP, Saifeddine M, Mihara K, et al. Metformin Prevents Hyperglycemia-Associated, Oxidative Stress-Induced Vascular Endothelial Dysfunction: Essential Role for the Orphan Nuclear Receptor Human Nuclear Receptor 4A1 (Nur77). Mol Pharmacol. 2021;100(5):428–455. doi: 10.1124/molpharm.120.000148 34452975

[62] Mather KJ, Verma S, Anderson TJ. Improved endothelial function with metformin in type 2 diabetes mellitus. J Am Coll Cardiol. 2001;37(5):1344–1350. doi: 10.1016/s0735-1097(01)01129-9 11300445

